# The Quality Changes and Proteomic Analysis of Cattle Muscle Postmortem during Rigor Mortis

**DOI:** 10.3390/foods11020217

**Published:** 2022-01-13

**Authors:** Zhenjiang Ding, Qichao Wei, Chunmei Liu, Hong Zhang, Feng Huang

**Affiliations:** 1 Institute of Food Science and Technology, Chinese Academy of Agricultural Sciences/Key Laboratory of Agro-Products Processing, Ministry of Agriculture and Rural Affairs, Beijing 100193, China; dingzhj18@lzu.edu.cn (Z.D.); weiqichao72@163.com (Q.W.); liuchunmei980119@163.com (C.L.); tfpmlab@163.com (H.Z.); 2 State Key Laboratory of Applied Organic Chemistry and College of Chemistry and Chemical Engineering, Lanzhou University, Lanzhou 730000, China

**Keywords:** rigor mortis, proteomics, meat quality, bioinformatics, protein biomarkers

## Abstract

Rigor mortis occurs in a relatively early postmortem period and is a complex biochemical process in the conversion of muscle to meat. Understanding the quality changes and biomarkers during rigor mortis can provide a theoretical basis for maintaining and improving meat quality. Herein, a tandem mass tag proteomic method is used to investigate the effects of differentially expressed proteins on the meat quality of cattle *Longissimus lumborum* muscle postmortem (0, 6, and 24 h). The pH, total sulfhydryl content and sarcomere length decrease significantly during storage. In contrast, meat color values (L*, a*, and b*) and the myofibril fragmentation index increase significantly. Altogether, 147 differentially expressed proteins are identified, most being categorized as metabolic enzymes, mitochondrial proteins, necroptosis and ferroptosis proteins and structural proteins. The results also reveal additional proteins that are potentially involved in rigor mortis, such as cardiac phospholamban, acetyl-coenzyme A acyltransferase, and ankyrin repeat domain 2. The current results provide proteomic insights into the changes in meat quality during rigor mortis.

## 1. Introduction

Tenderness is an important indicator affecting both meat quality and the purchase intention of consumers. Decreased tenderness (the opposite of tenderness) may be due to background toughness and myofibrillar toughness [1]. Myofibrillar toughness is affected by three phases: the prerigor phase, rigor phase and tenderization phase [2]. Rigor mortis, including delayed phase and rapid phase, is one of the most important and complex biochemical processes in the conversion of muscle to meat. It occurs in a relatively early postmortem (PM) period, resulting in an increase in the toughness of the meat [3]. Even though the blood circulation that supplies oxygen and removes the end products of metabolism is exhausted, the muscles still maintain function and metabolic activity. The sole purpose of anaerobic metabolism of glycogen and high-energy phosphate compounds is to produce adenosine triphosphate (ATP) in the early postmortem muscles. Lactate and hydrogen ions (H^+^) are the end products of glycolysis and ATP hydrolysis, respectively. They accumulate in muscles due to the lack of an effective elimination mechanism, leading to a decrease in pH [4]. This acidification leads to the reduction of water-holding capacity (WHC) and the release of calcium, resulting in the formation of a cross bridge between myosin and actin filaments [5]. In addition, as the glycogen level in the muscle decreases after slaughter, the available energy to maintain the relaxed state of the muscle is also reduced. The combination of these factors results in the onset and development of rigor mortis [6]. Accordingly, it is particularly important to illuminate the complex biochemical mechanism of rigor mortis for the preservation of meat after slaughter and the improvement of the profitability of the meat industry.

Proteomics can be defined as the science of studying a subset of all proteins expressed in a certain cell or tissue and has been employed in meat science to explore molecular mechanisms occurring in muscle and affecting meat quality [7]. Proteomic research on beef is the first step to understanding the mechanism of the conversion from muscle to meat and the control of meat quality. The effects of complex proteolysis and the interaction of soluble muscle proteins in aging on meat quality remain major challenges. Finding new protein biomarkers that regulate muscle growth would help scientists test better experimental hypotheses, enabling the meat production industry and consumers to obtain better quality meat [8]. In recent years, scientists have discovered some metabolic enzymes, heat shock proteins (HSPs), oxidative proteins, structural proteins, and proteases related to meat tenderness, color, and WHC as protein biomarkers through proteomics. Metabolic enzymes including glyceraldehyde-3-phosphate dehydrogenase (GAPDH), glycogen phosphorylase (PYGM), pyruvate kinase isozymes M2 isoform (PKM2), adenylate kinase isoenzyme 1 (AK1), fructose-bisphosphate aldolase A isoform (ALDOA), triosephosphate isomerase (TPI1), retinal dehydrogenase 1 (ALDH1A1), malate dehydrogenase (MDH1), and lactoylglutathione lyase (GLO1) are closely correlated with meat color and pH in *M longissimus lumborum* during PM storage [5,9,10]. Similarly, beta-enolase (ENO3), phosphoglucomutase-1 (PGM1), protein-L-isoaspartate O-methyltransferase (PCMT1), and proteasome subunit beta type-2 (PSMB2) are correlated with the pH and a* value of meat from *semitendinosus* by Pearson’s correlation analysis [11]. In addition, HSPB1, αβ-crystallin, HSP40, HSP70-8 and HSP90 belong to the family of HSPs that can regulate meat quality [9,12,13]. Peroxiredoxin-1 (PRDX1), peroxiredoxin-2 (PRDX2), peroxiredoxin-6 (PRDX6), superoxide dismutase (SOD), and DJ-1 (PARK7) have a key role in the degradation of peroxide and show an increase in oxidative damage in the tender meat group compared to the intermediate and tough meat groups [14]. Structural proteins, including α-actin, titin, and myosin heavy chain (MyHC), were of great significance to discriminate the meat color classes [15]. Myosin regulatory light chain 2, keratin, and desmin significantly varied between low- and high-drip groups in the *pectoralis major* of geese [13]. In the process of meat aging, proteases such as calpains, caspases, lysosomal cathepsins, and proteasomes hydrolyze structural proteins to increase the tenderness of meat [16]. Of the proteins summarized above, it is possible that one protein regulates multiple characteristics of meat quality, and that there are interactions between proteins to regulate meat quality. At present, the application of proteomics is mainly focused on the aging of muscle PM. However, there are few studies focusing on protein changes in rigor mortis, and new protein biomarkers need to be explored to supplement the mechanism of rigor mortis in the process of converting from muscle to meat.

The objective of this study is to investigate protein changes in rigor mortis. Tandem mass tags (TMTs) for quantitation analysis of *longissimus lumborum* (LL) muscle from cattle with different rigor mortis times were executed. It is hoped that the data will provide a new perspective for the improvement of biochemical mechanisms, discovery of potential protein biomarkers in rigor mortis, and subsequent exploration of the mechanism of meat quality changes.

## 2. Materials and Methods

### 2.1. Animals and Muscle Sampling

**Figure 1:**
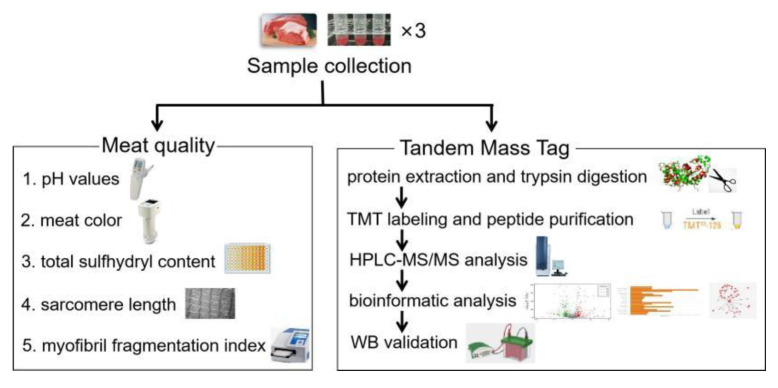
Experimental design and workflow. TMT: tandem mass tags, HPLC-MS/MS: liquid-chromatography tandem mass spectrometry, WB: western blot.

Sixty improved cattle (Simmental × Chinese Luxi yellow cattle, an average living weight of 400 ± 50 kg, approximately 8 months old) were slaughtered on the same day by a commercial meat processing company (Beijing Zhuochen Animal Husbandry Co., Ltd., Beijing, China) without electrical stimulation in accordance with National Standards of China (GB/T 17237-2008; date: 2018). The protocol was approved by the Animal Care and Ethics Committee of Chinese Academy of Agricultural Sciences (IAS20160616; date: 2016, Beijing, China). The experimental design was summarized in Figure 1. The LL muscle was taken immediately from the carcasses within 30 min. Approximately 10 g muscle (approximately 2 cm × 2 cm × 2 cm, the sample mass for one repetition) was cut and measured for pH immediately. Another 20 g of muscle was incubated in sterilized physiological saline solution for 6 and 24 h at 4 °C to prevent the growth of microorganisms in the air and facilitate subsequent research. The muscle was subjected to measurements of color, myofibril fragmentation index (MFI), sarcomere length, total sulfhydryl content, and total protein extraction at the sampling time after the measurement of initial pH. 

### 2.2. pH Values

The pH values were detected at 0, 6, and 24 h PM at 4–6 random locations (repeat one time per 10 g muscle) with a portable pH meter equipped with a penetration probe (Testo205 pH meter, Lenzkirch, Germany). The pH meter allowed for the automatic detection of buffer solutions and automatic temperature compensation. The probe of the pH meter was inserted directly into the meat about 1 cm deep from the meat surface, and the values averaged from 4–6 different points were taken as the final measured pH values.

### 2.3. Meat Color 

The meat color values CIE L* (lightness), a* (redness), and b* (yellowness) were measured at 0, 6, and 24 h PM at 4–6 random locations (repeat one time per 10 g muscle) using a colorimeter (CM-600D, Osaka, Japan). The colorimeter was calibrated with a white tile (Y = 93.6, x = 0.3130, y = 0.3193) specific to the machine before measuring each sample and set to an illuminant/observer angle of D-65/2°, aperture size of 8 mm, and measurement time of 1 s. The muscle was cut (approximately 2 cm × 2 cm × 2 cm) into two pieces from the middle, and meat color values were measured on the freshly cut surface after 30 min of blooming at room temperature.

### 2.4. Total Sulfhydryl Content

Total protein extraction and total sulfhydryl content were measured according to our published work [12]. Total proteins were extracted with phosphate buffer containing 2% sodium dodecyl sulfate (SDS) and adjusted to a uniform protein concentration using the Bradford assay. The total sulfhydryl content was measured with 1 mM 5,5′-Dithiobis-(2-nitrobenzoic acid) (DTNB) (6 M guanidine hydrochloride, pH 8.0) by reading the absorbance at 412 nm with a microplate reader (BioTek, Winooski, VT, USA). The residual total proteins were subjected to proteomics.

### 2.5. Sarcomere Length

Transmission electron microscopy (TEM) was performed according to Zhang et al. [17] with some modifications. The meat samples with a size of 2 mm × 2 mm × 4 mm were fixed with glutaraldehyde (2.5%) for 24 h, washed with phosphate buffer (pH = 7.2), dehydrated by an ethanol gradient (30%, 50%, 70%, 90%, and 100%), and exchanged in acetone. The samples were then embedded in epoxy resin and sliced using a Leica EM UC6 microtome (Leica, Solms, Germany). Ultrathin sections were stained with uranyl acetate and lead citrate and observed using a New Bio-TEM H-7500 TEM (Hitachi, Tokyo, Japan). The sarcomere length was calculated in the micrographs created using Image-Pro Plus 6.0 software (Media Cybernetics, Rockville, MD, USA).

### 2.6. Myofibril Fragmentation Index(MFI)

The MFI was determined according to Zhang et al. [17] with minor modifications. The minced muscle (0.5 g), trimmed of visible fat and connective tissue, was homogenized in 5 mL of ice-cold buffer (100 mM KCl, 1 mM MgCl_2_, 20 mM K_2_HPO4, and 1 mM EDTA; pH = 7.0). The samples were centrifuged for 15 min at 3000× *g*. The precipitate obtained was resuspended in 5 mL of ice-cold buffer and centrifuged again. The precipitate was then resuspended in buffer and filtered with a polyethylene strainer (20 mesh). The protein concentration of the filtrate was measured by the biuret method, and the concentration was then adjusted to 0.5 ± 0.05 mg/mL with buffer. The absorbance at 540 nm was measured and the result was multiplied by 200 to obtain the MFI value.

### 2.7. Trypsin Digestion and Tandem Mass Tag (TMT) Labeling

Each sample (approximately 100 μg of total proteins) was precipitated with 4 vol (*v*/*v*) of ice-cold acetone, and the precipitate was resuspended in reducing buffer (8 M urea, 30 mM HEPES, 1 mM PMSF, 2 mM EDTA, 10 mM DTT) and incubated at 56 °C for 1 h. Then, iodoacetamide (55 mM) was added to alkylate in the dark for 45 min. The mixture was centrifuged in a 10 kDa ultrafiltration tube for 20 min at 12,000 rpm and 4 °C, and the filter was washed twice with 200 μL of 50 mM TEAB and centrifuged at 12,000 rpm for 20 min. The proteins were digested with 3 μg trypsin/100 μg proteins overnight at 37 °C. The resulting peptides were lyophilized and then further reconstituted in 30 μL TEAB for labeling. Each TMT reagent was dissolved in 70 μL of isopropanol mixed with the peptide solution [18].

### 2.8. Peptide Purification and HPLC-MS/MS Analysis

The labeled peptide was diluted with solution A (0.1% formic acid, 2% ACN, 98% water) and preseparated with solution B (0.1% formic acid, 2% water, 98% ACN), and different components were collected at different times. The collected peptides were desalted and purified by C18 column according to the manufacturer’s instructions. Desalted peptide mixtures were loaded onto an Acclaim PepMap C18 reversed-phase column (75 μm × 2 cm, 3 μm) and separated with a reversed-phase C18 column (75 μm × 10 cm, 5 μm) mounted on a Dionex UltiMate 3000 Nano LC system. Peptides were eluted using a gradient of solution B (5%, 5%, 30%, 60%, 80%, 100%) at a flow rate of 300 nL/min combined with a Q Exactive mass spectrometer (Thermo Fisher Scientific, MA, USA). The eluates were directly entered into the Q Exactive MS (Thermo Fisher Scientific, Waltham, MA, USA) in positive ion mode and data-dependent manner with a full MS scan from 350–2000 m/z, full scan resolution at 70,000, and MS/MS scan resolution at 17,500. An MS/MS scan with minimum signal threshold 1 × 10^5^, isolation width at 2 Da was completed. The MS/MS acquisition mode was higher collision energy dissociation (HCD). To optimize the MS/MS acquisition efficiency of HCD, normalized collision energy (NCE) was systemically examined with a stepping of 20%.

### 2.9. Data Process and Bioinformatic Analysis

The obtained MS/MS data were analyzed using Proteome Discoverer 1.4 and searched using Mascot version 2.3.0. Information for the UniProt database and the reversed database was collected as follows: database, UniProt-bovine (ID: 9913); peptide mass tolerance, 15 ppm; MS/MS tolerance, 20 mmu; max missed cleavages, 1; fixed modification, carbamidomethyl (C); variable modification, oxidation (M), Gln→pyro-Glu (N-term Q), TMT 6 plex (K), TMT 6 plex (N-term); protein ratio type, median; minimum peptides, 1; normalization method, median. Based on the identified proteins, ANOVA was applied to screen out the differences in protein expression among the three groups (0, 6 and 24 h PM) with *p* < 0.05 and fold change > 1.5 or <2/3. Then, RStudio and Cytoscape were used to perform bioinformatic analysis of the obtained differential proteins, including gene ontology (GO, http://geneontology.org/, accessed on 10 July 2021), the Kyoto Encyclopedia of Genes and Genomes (KEGG, http://www.genome.jp/kegg/, accessed on 10 July 2021) pathway, and the protein–protein interaction (PPI, https://string-db.org/, accessed on 10 July 2021) network [18]. The significance level of the protein enrichment of a given GO term was tested by Fisher’s exact test. The significance level of protein enrichment in each pathway by Fisher’s exact test in the unit of the KEGG pathway was obtained to identify the significant metabolic and signal transduction pathways. Regarding PPI, default settings were used, i.e., medium confidence of 0.4 and 4 criteria for linkage: co-occurrence, experimental evidence, existing databases, and text mining.

### 2.10. SDS-PAGE and Western Blotting

To further check the changes in protein expression levels, SDS-PAGE and western blotting analysis, as well as additional quantification, were performed according to our published work [12]. The expression levels of pyruvate kinase (PKM), troponin T (TNNT1), cytochrome c oxidase subunit 5A (COX5A), mitochondrial fission 1 protein (FIS1), cytochrome c oxidase subunit 4 isoform 1 (COX4I1), and NADH dehydrogenase [ubiquinone] 1 beta subcomplex subunit 11 (NDUFB11) were detected through SDS-PAGE and western blotting. Total proteins were extracted according to our previous study [12]. The minced muscle was homogenized in 1.5 mL of ice-cold 2% SDS in 10 mM phosphate buffer (pH 7.2) and quantified using the Bradford assay. 12.5% polyacrylamide separating gels and 4.5% polyacrylamide stacking gels were used, and 40 μg samples were loaded per well. The gels were electrophoresed on the Bio-Rad Mini-PROTEAN II system (Bio-Rad Laboratories, Hercules, CA, USA) at a voltage of 80 V for 0.5 h, followed by 120 V until the indicator line reached the bottom of the gel. After electrophoresis, proteins were transferred to a membrane at 200 mA and 4 °C for 60 min and then blocked with 5% (*w*/*v*) nonfat dry milk in TBST (20 mM Tris, 137 mM NaCl, 5 mM KCl, and 0.05% (*v*/*v*) Tween 20), and the membrane was then incubated with anti-PKM2 (D220008, Sangon Biotech, Shanghai, China, 1:1000), anti-troponin T (clone JLT-12, Sigma, Saint Louis, MO, USA, 1:2000), anti-COX5A (ab110262, Abcam, Cambridge, UK, 1:2000), anti-FIS1 (D222377, Sangon Biotech, Shanghai, China, 1:1000), anti-COX4I1 (ab16056, Abcam, Cambridge, UK, 1:2000), and anti-NDUFB11 (D223630, Sangon Biotech, Shanghai, China, 1:1000) according to the manufacturers’ instructions. The membranes were incubated with secondary antibodies (A9044, Sigma, Saint Louis, MO, USA, 1:4000) after washing four times with TBST buffer, and then the proteins were visualized by immobilon western chemilum HRP (ECL) substrate (Bio-Rad, Hercules, CA, USA) after washing four times with TBST buffer. The membranes were imaged by ChemiDocTMMP Imaging System (Bio-Rad, Hercules, CA, USA).

### 2.11. Statistical Analysis

The significant differences among different PM storage times were analyzed by the SPSS statistical software package (17.0, IBM, Chicago, IL, USA). LSD and Duncan’s multiple range test were used to compare individual mean differences (*p* < 0.05). The data in all experiments were expressed as the mean ± SD (standard deviation) from 4–6 replicates. The densities of targeted bands were analyzed using Quantity One software (Bio-Rad, Hercules, CA, USA). The protein densities at 0 h were used as the control when comparing the differentially expressed proteins at 0 h vs. 6 h PM. Protein densities of 6 h were used as the control when comparing the differentially expressed proteins of 6 h vs. 24 h PM. Statistical differences between the two groups were determined by Student’s t-test.

## 3. Results and Discussion 

### 3.1. pH and Meat Color

The pH values of skeletal muscle during rigor mortis are shown in Table 1. The pH values significantly decreased within 24 h PM. This may be due to the accumulation of lactate acid and H^+^, which are the final products of glycolysis and ATP hydrolysis, respectively [4]. The rapid decrease in the pH values PM affected not only the onset and development of rigor mortis [19] but also protein degradation and endogenous enzyme activity during aging [20], which in turn regulated meat tenderness. 

Meat color is the most intuitive indicator for consumers to evaluate meat product quality. The L* value of skeletal muscle increased significantly from 6 to 24 h, while there was no significant difference from 0 to 6 h (Table 1). The a* value of skeletal muscle continued to increase from 0 to 24 h after slaughter (*p* < 0.05) (Table 1). The b* value increased significantly from 0 to 24 h (Table 1). The sarcoplasmic protein degeneration and myofibril shrinkage that occurred in the early stage of PM increased the scattering of meat and caused meat to become lighter. In addition, myoglobin was oxygenated to oxymyoglobin when atmospheric oxygen permeated the meat surface, resulting in the meat appearing bright red [21].

### 3.2. Total Sulfhydryl Content, Sarcomere Length and MFI

As an important reactive group of proteins or small biological molecules in cells, sulfhydryl groups are oxidized to form disulfide bonds, sulfonic acid, and other oxidative derivatives by oxidizing agents produced during meat storage, resulting in meat quality deterioration [12]. Therefore, the oxidation state has an important biological function for molecular activity. Total sulfhydryl content was measured found to gradually decrease during rigor mortis (Table 2). Total sulfhydryl content is a good indicator of protein oxidation level. A study of refrigerated turkey meat revealed similarly reduced sulfhydryl content during storage [14].

**Figure 2:**
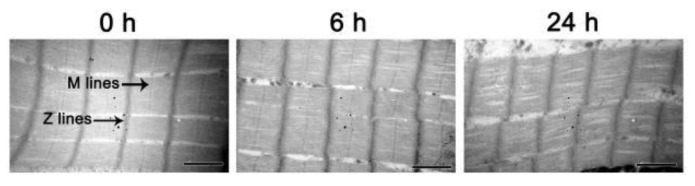
Transmission electron microscopy (TEM) images of *longissimus lumborum* muscles during postmortem storage.

Proteolysis after slaughter can lead to ultrastructural changes of myofibrils, including Z-lines, M-lines, and A-bands, which are related to the tenderization of muscle into meat [22]. TEM images of myofibrils PM are shown in Figure 2. The apparent Z-lines and M-lines were clearly perceived, and the sarcomere structure was neatly arranged in the 0 h sample. Some misalignment of the sarcomeres was perceived in the 6 h PM samples. The ultrastructure of the muscle at 24 h after slaughter showed obvious changes compared with 0 h, mainly reflected in the obscured Z-lines and M-lines and the complete misalignment of the sarcomere structure. The destruction of the sarcomere structure may be caused by changes in the interaction of F-actin, actomyosin, and tropomyosin [23]. In addition, sarcomere length, determined from the TEM images, was shown in Table 2. The sarcomere length of skeletal muscle decreased significantly from 0 to 24 h PM. Similar results were reported by Wang et al. [24], who concluded that the sarcomere length of the *Longissimus lumborum*, *semimembranosus* and *Psoas major* muscles evidently decreased from 0.5 to 24 h PM. The sarcomere length of breast muscles from reared broilers decreased significantly until 3 h PM [25]. This may be caused by the formation of cross-links between myosin S-1 heads and actin, resulting in actomyosin in rigor mortis [26]. Additionally, the decrease in sarcomere length may also be related to cold shortening caused by diminished functioning of the calcium pumps in the sarcoplasmic reticulum at low temperatures [27].

MFI is a useful index that reflects the degree of proteolysis, thereby characterizing tenderness, indicating I-band breakage and intermyofibril linkages [17]. As shown in Table 2, MFI increased significantly with the increase of time PM, which is consistent with the previous result that MFI of meat from breast and thigh increased significantly 12 h PM [17]. The overlap of myosin filaments from one sarcomere with actin filaments to the next may form a continuously linked actomyosin after formation of rigor crossbridges. At very short sarcomere lengths, myofibrils within a sarcomere can be ruptured by the shortening of neighboring sarcomeres [26].

### 3.3. Bioinformatics Analysis of the Differential Proteins and WB Validation

Proteome analysis based on tandem mass tags (TMTs) has been widely used in meat science due to its proteome coverage, accurate quantification, and high technical reproducibility [5]. According to TMT analysis, 83 differentially expressed proteins were identified between 6 h and 0 h PM, including 36 upregulated and 47 downregulated proteins, which are summarized in Table S1. In total, 71 proteins were determined to be different in abundance between 24 h and 6 h PM, of which 12 proteins were upregulated and 59 were downregulated in the 24 h PM group, as summarized in Table S2. 

GO annotation, consisting of biological processes, cellular components, and molecular functions, was performed to obtain information about the mechanism of rigor mortis. The top 10 specific items of the three categories of differential proteins between 6 h and 0 h PM are shown in Table 3. For biological processes, ADP (adenosine diphosphate) metabolic process (GO:0046031), ATP generation from ADP (GO:0006757), nucleoside diphosphate metabolic process (GO:0009185), ribonucleoside diphosphate metabolic process (GO:0009185), pyruvate biosynthetic process (GO:0042866), and other biological processes related to metabolism were significantly enriched. Cellular components were enriched in contractile fiber (GO:0043292), I band (GO:0031674), myofibril (GO:0030016), and sarcomere (GO:0030017), which are related to myofibrillar proteins and sarcomere structure. For molecular function, the major classes of proteins were magnesium ion binding (GO:0000287), superoxide dismutase activity (GO:0004784), and transferase activity (GO:0016740). From each of the three categories of differential proteins, the 10 most-observed proteins between 24 h and 6 h PM are shown in Table 4. For biological processes, drug transport (GO:0015893), establishment of localization (GO:0051234), ion transport (GO:0006811) and other biological processes related to transport were significantly enriched. Cellular components were mainly enriched in various membrane parts, such as the integral component of membrane (GO:0016021), intrinsic component of membrane (GO:0031224), mitochondrial membrane (GO:0031966), and organelle membrane (GO:0031090). For molecular function, the major classes of these proteins were active transmembrane transporter activity (GO:0022804), inorganic molecular entity transmembrane transporter activity (GO:0015318), and ion transmembrane transporter activity (GO:0015075). These GO analyses of the differentially expressed proteins of 6 h vs. 0 h and 24 h vs. 6 h are displayed in Tables S3 and S4, respectively.

KEGG pathway enrichment was performed to clarify the biological pathways involved in the differentially expressed proteins during rigor mortis. The top 20 specific differential proteins between 6 h and 0 h PM are shown in Table 5, including biosynthesis of amino acids (map01230), carbon metabolism, glycolysis/gluconeogenesis (map00010), insulin signaling pathway (map04910), and pyruvate metabolism (map00620). The top 13 significantly enriched pathways of differential proteins between 24 h and 6 h PM were selected (Table 6), including the calcium signaling pathway (map04020), cGMP-PKG signaling pathway (map04022), necroptosis (map04217), and oxidative phosphorylation (map00190). These KEGG pathway analyses of the differentially expressed proteins of 6 h vs. 0 h and 24 h vs. 6 h are displayed in Tables S5 and S6, respectively. These biological processes may involve the onset and development of rigor mortis.

**Figure 3:**
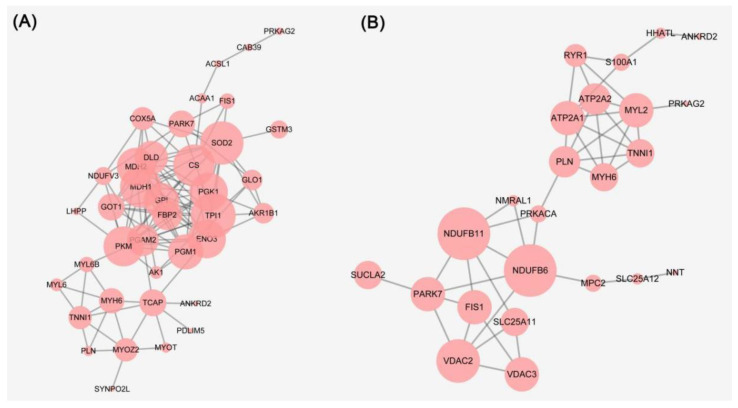
(**A**) Protein-protein interaction networks of differentially expressed proteins of 6 h vs. 0 h PM. (**B**) Protein-protein interaction networks of differentially expressed proteins of 24 h vs. 6 h PM.

The PPI network of the differentially expressed proteins of 6 h vs. 0 h and 24 h vs. 6 h was constructed and shown in Figure 3A,B, respectively. The proteins at 6 h vs. 0 h that were pyruvate kinase (PKM), dihydrolipoyl dehydrogenase (DLD), phosphoglycerate mutase 2 (PGAM2), fructose-1,6-bisphosphatase isozyme 2 (FBP2), phosphoglycerate kinase 1 (PGK1), glucose-6-phosphate isomerase (GPI), beta-enolase (ENO3), triosephosphate isomerase (TPI1), lactoylglutathione lyase (GLO1), and malate dehydrogenase (MDH1/2). These proteins were implicated in glycolysis/gluconeogenesis, pyruvate metabolism, carbon metabolism, citrate cycle, and oxidative phosphorylation. The proteins at 24 h vs. 6 h were cardiac phospholamban (PLN), ryanodine receptor 1 (RYR1), calcium-transporting ATPase (ATP2A2), cAMP-dependent protein kinase catalytic subunit alpha (PRKACA), voltage-dependent anion-selective channel protein 2 (VDAC2), sarcoplasmic/endoplasmic reticulum calcium ATPase 1 (ATP2A1), voltage-dependent anion-selective channel protein 3 (VDAC3), myosin heavy chain 6 (MYH6), myosin regulatory light chain 2 (MYL2), and protein kinase AMP-activated non-catalytic subunit gamma 2 (PRKAG2). These proteins were implicated in the calcium signaling pathway, cGMP-PKG signaling pathway, ferroptosis, necroptosis, tight junction, cardiac muscle contraction, vascular smooth muscle contraction, and focal adhesion. 

**Figure 4:**
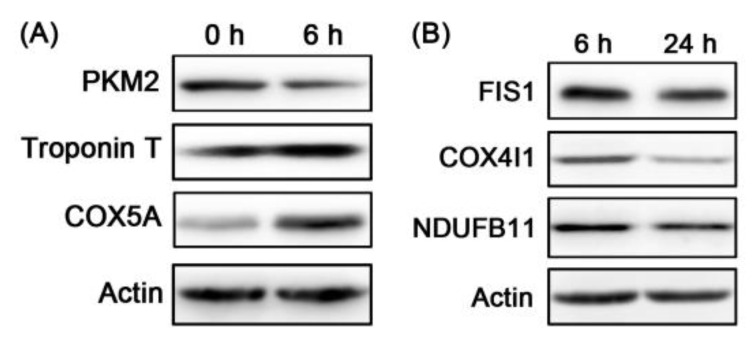
(**A**) Validation of the differentially expressed proteins of 0 h vs. 6 h PM by western blotting. (**B**) Validation of the differentially expressed proteins of 6 h vs. 24 h PM by western blotting.

Several different upregulated and downregulated proteins were selected to validate the TMT results by western blotting, including pyruvate kinase (PKM), troponin T (TNNT1), cytochrome c oxidase subunit 5A (COX5A), mitochondrial fission 1 protein (FIS1), cytochrome c oxidase subunit 4 isoform 1 (COX4I1), and NADH dehydrogenase [ubiquinone] 1 beta subcomplex subunit 11 (NDUFB11). As shown in Figure 4 and Table 7, the changes in these protein levels were consistent with the results of TMT analysis, indicating that the TMT results in this study are reliable.

### 3.4. Metabolic Enzymes

The changes in metabolic enzymes after slaughter were significantly related to the pH and meat color in rigor mortis [18,20]. PKM2, DLD, PGAM2, GAPDH, PGM, FBP2, PGK1/2, GPI, ENO3, and TPI1 are all involved in the glycolysis/gluconeogenesis pathway, and were detected in the differential proteins of samples between 0 h and 6 h after slaughter in this study. In particular, PKM2 is a key rate-limiting enzyme for glycolysis, catalyzing the transfer of the phosphate group of phosphoenolpyruvate (PEP) to ADP to generate pyruvate and ATP. Higher PKM2 activity is a sign of faster glycolytic metabolism, and various posttranslational modifications (phosphorylation, acetylation, oxidation, and methylation) affect PKM2 activity [28]. Phosphorylation levels of PKM2 at Thr155 were significantly different under different glycolysis rates in ovine muscles [29]. TPI1 is an important glycolytic enzyme that catalyzes the mutual conversion of dihydroxy-acetone phosphate and glyceraldehyde 3-phosphate to provide energy for muscle cells. It has been proposed as a potential biomarker to regulate the development and stability of beef color and pH during storage [30]. TPI1, including its phosphorylated form, has an effect on the accumulation of lactate and hydrogen protons, which may regulate the level of electrostatic rejection of myofibrillar and filament proteins, thereby affecting the transverse contraction of muscle fibers and lengthening of sarcomeres [5]. In addition to some of the aforementioned proteins, GLO1 and MDH were detected in the pyruvate metabolism pathway. MDH, as a component of the tricarboxylic acid cycle, catalyzed the reaction that regenerates oxaloacetate and was related to the malate–aspartate shuttle system through the mitochondrial membrane to transport reductants. The L-lactate dehydrogenase (LDH)/MDH ratio was used to reflect the oxidation level of muscle, and the lower ratio was similar to active oxidative metabolism [31]. 

### 3.5. Mitochondrial Electron Transport Chain 

Protein subunits of complex I (NDUFB6 and NDUFB11), complex II (SDHC and SDHD), complex IV (COX2, COX4I1, COX7C, and COX6C), and complex V (ATP5PO and ATP5MF) of the mitochondrial electron transport chain were detected in the differentially expressed proteins of 24 h vs. 6 h PM. The results of Beldarrain et al. [21] suggested that the electrons located between complex III and IV may reduce metmyoglobin. In addition, complex I and complex II regulated the production of NADH, which can be used for the reduction of metmyoglobin through electron transport mediation and enzymatic pathways, thereby affecting meat color [32]. Complex V converted ADP into ATP under the premise of a proton gradient, and the degradation degree of complex V was directly related to the ATP/ADP homeostasis and affected the stability of meat color [12]. 

### 3.6. Necroptosis and Ferroptosis

As a new type of cell death mode, necroptosis was first proposed by Degterev et al. [33], and it has the characteristics of both apoptosis and necrosis. Histone H2A type 2-C (H2AC20) and ADP/ATP translocase 1 (SLC25A4) belong to the necroptosis pathway and were detected in the differentially expressed proteins of 24 h vs. 6 h PM. SLC25A4 participated in muscle contraction and the calcium-mediated signaling pathway and was abundant in transcription in muscle tissue from two pig breeds [34]. Ferroptosis is a unique iron-dependent form of nonapoptotic cell death caused by the lethal small molecule erastin [35]. VDAC2 and VDAC3 belong to the ferroptosis pathway and were detected in the differentially expressed proteins of 24 h vs. 6 h PM. VDAC3 participated in the ferroptosis pathway characterized by iron-dependent lipid peroxidation accumulation and regulated cell death in *longissimus dorsi* muscle from Xidu black pigs [36]. Ubiquitination of VDAC2/3 by Nedd4 inhibited ferroptosis in melanoma induced by erastin [37]. Interestingly, SLC25A4 interacted with VDAC2, VDAC3, and NDUFB11 (Figure 3). NDUFB11 participated in mitochondrial function and was associated with apoptosis, indicating that there is a certain correlation between necroptosis, apoptosis, and ferroptosis. Similarly, cytochrome c was released from the mitochondria into the cytoplasm, leading to the activation of caspases, which can regulate the adenine nucleotide translocator encoded by SLC25A4 and VDAC1/2 [38].

### 3.7. Structural Proteins 

The degradation of myofibrillar proteins was related to tenderness, and more fragments than intact structures were observed in tender meat [12]. Some structural proteins identified in this study are known to be related to tenderness, including troponin (TNNC1, TNNT1, and TNNT3), myosin (MYL2, MYL6, MYL6B), and keratin (KRT10, KRT14). Troponin is a heterotrimeric complex composed of troponin T, troponin I, and troponin C subunits, and plays a central regulatory role in the process of striated muscle contraction [12]. Calcium ions bound to the nh2 domain of troponin C, promoting its interaction with troponin I, leading to its dissociation from actin during muscle contraction. Finally, the troponin I-troponin T complex anchored to the tropomyosin-actin filaments and enhanced actomyosin bonds [21]. Thus, degradation of troponin means that the interacting proteins are destroyed, and the thin filaments in the sarcomeric I band may be broken. The myosin family depended on ATP to participate in muscle contraction and various intracellular functions, such as cell migration and adhesion, signal transduction, intracellular transportation, and localization of macromolecules [11]. Myosin light chains (MYL3 and MYL6B) and regulatory light chain 2 isoforms (MYL2 and MYLPF) were involved in the conversion of muscle to meat, and myosin was a major structural protein related to actin and other contractile proteins. Denaturation of the head of myosin can cause myofibrils to shrink laterally and reduce the WHC [39]. Keratin, as a kind of myofibril protein, was found to be a differentially expressed protein between high and low drip loss groups from geese, which was related to WHC [13].

### 3.8. Potential Markers during Rigor Mortis 

In addition to the aforementioned proteins, some distinctly different proteins were detected in this study, which may serve as markers that affect the onset and development of rigor mortis. PLN was detected in the differentially expressed proteins of 24 h vs. 6 h PM and participated in some pathways related to calcium ions (calcium signaling pathway and cGMP-PKG signaling pathway) and cardiac contraction and relaxation (adrenergic signaling in cardiomyocytes, thyroid hormone signaling pathway, and dilated cardiomyopathy). PLN controlled the storage level of sarcoplasmic reticulum calcium ions in human hearts and can also act as a regulatory protein for adrenaline. Calcium ion transportation in cells could be affected by mutations of PLN, leading to abnormal myocardial function. Similar to troponin T, PLN is considered a candidate gene for dilated cardiomyopathy because of its role in myofibril calcium sensitivity [40]. In addition, acetyl-coenzyme A acyltransferase (ACAA) includes two subtypes: ACAA1 and ACAA2. ACAA1 was detected in the differentially expressed proteins and mainly involved in physiological and biochemical processes, such as long-chain fatty acid oxidation, bile acid metabolism, and regulation of peroxisome proliferation [41]. The study has shown that increasing ACAA activity can promote peroxisomal fatty acid β-oxidation, and the accumulation of oxidized polyunsaturated fatty acids is the main cause of ferroptosis [35]. Ankyrin repeat domain 2 (ANKRD2), which belongs to the muscle ankyrin repeat protein (MARP) family, was detected among the differentially expressed proteins. The MARP family plays a key role in the integration of cytoskeletal structure, communication between the sarcoplasm and the nucleus, and stress response [42]. ANKRD2 is a skeletal muscle protein involved in skeletal muscle hypertrophy and located in the I band [43]. The expression profile of porcine ANKRD2 indicated that it may be related to meat quality regulated by muscle fiber type [44]. Moreover, the differential proteins obtained in this study still contained a small number of unknown proteins. These proteins may also play an important role in the rigor mortis of beef, and further research is needed.

### 3.9. Meat Quality and Differentially Expressed Proteins

Glycolysis is an important biochemical pathway affecting meat quality in PM muscles [5]. Glycolytic enzymes, including PKM2 (6 h vs. 0 h), TPI1 (6 h vs. 0 h), ENO3 (6 h vs. 0 h) and LDH-B (24 h vs. 6 h) were detected in the differential proteins, and the expression levels were basically downregulated to half. Rosenvold et al. revealed that these proteins led to changes in pH [45]. Consumers regard meat color as the main criterion to judge whether meat is fresh or not. Both hemoglobin and myoglobin are the main pigment proteins, and the key substances that determine meat color [46]. Interestingly, downregulation of hemoglobin in the differential proteins between 6 h and 24 h PM was detected in this study. Moreover, downregulation of NDUFB6, NDUFB11, SDHC, SDHD, COX2, COX4I1, COX7C, COX6C, ATP5PO, and ATP5MF in the differential proteins between 6 h and 24 h PM were detected. They can be classified as belonging to complex I, II, IV, and V, which were involved in the mitochondrial electron transport chain. Previous research suggested that the complex can participate in the regulation of myoglobin, thereby affecting the meat color [29,47]. GAPDH, PRDX6, PGM1, and SOD detected in the differential protein between 0 h and 6 h were potential predictors of meat color traits, consistent with the results of Wu et al. [48]. Thus, metabolic enzymes, oxidative proteins and mitochondrial electron transport chain complex may be involved in the regulation of meat color at different stages of rigor mortis. Additionally, the change in sarcomere length and MFI may depend primarily on the structural proteins in the differential proteins. Notably, as a new type of cell death mode, ferroptosis has received enthusiastic attention from scientists. VDAC2 and VDAC3 proteins involved in ferroptosis were detected in the differential proteins in this study. ACAA1 was also speculated to participate in ferroptosis because of its function on the regulation and accumulation of long-chain fatty acid oxidation. This provides a new perspective for the study of the mechanism of ferroptosis in the regulation of meat quality.

## 4. Conclusions

The quality and protein changes of PM meat used to elucidate the biochemical mechanism of rigor mortis were explored. The beef meat quality including pH, meat color, total sulfhydryl content, sarcomere length, and MFI changed significantly within 24 h after slaughter. A total of 147 differentially expressed proteins involved in a certain number of signaling pathways were analyzed using TMT quantitative proteomics. Several proteins that may play a key role in rigor mortis were also proposed. Further verification of differential proteins and new strategies are needed to supplement the presented theories in the development of rigor mortis.

## Figures and Tables

**Table 1:** pH and meat color of *Longissimus lumborum* muscles during postmortem storage.

	pH	L*	a*	b*
0 h	6.541 ± 0.185 ^a^	29.066 ± 1.299 ^a^	8.650 ± 1.103 ^a^	4.864 ± 0.291 ^a^
6 h	5.530 ± 0.164 ^b^	29.744 ± 1.448 ^a^	11.294 ± 0.865 ^b^	7.156 ± 1.052 ^b^
24 h	5.224 ± 0.015 ^c^	34.429 ± 1.643 ^b^	13.997 ± 2.040 ^c^	9.888 ± 1.429 ^c^

L*, a*, and b* represent lightness, redness, and yellowness, respectively; different letters (^a–c^) in the same column indicate significant differences at different postmortem storage times (*p* < 0.05). The data were expressed as the mean ± SD.

**Table 2:** Total sulfhydryl content, MFI (myofibril fragmentation index), and sarcomere length of *Longissimus lumborum* muscles during postmortem storage.

	Total Sulfhydryl Content (%)	MFI	Sarcomere Length (μm)
0 h	100.00 ± 5.136 ^a^	52.233 ± 0.463 ^a^	1.278 ± 0.013 ^a^
6 h	80.058 ± 2.362 ^b^	56.333 ± 0.689 ^b^	1.164 ± 0.020 ^b^
24 h	67.928 ± 5.136 ^c^	61.000 ± 0.253 ^c^	1.054 ± 0.035 ^c^

Different letters (^a–c^) in the same column indicate significant differences at different postmortem storage times (*p* < 0.05). The data were expressed as the mean ± SD.

**Table 3:** Gene ontology classification results of differentially expressed proteins of 6 h vs. 0 h PM.

	Term	Description	*p* Value
Biological Process	GO:0046031	ADP metabolic process	6.10 × 10^−^^9^
	GO:0006757	ATP generation from ADP	3.02 × 10^−^^8^
	GO:0009132	Nucleoside diphosphate metabolic process	1.02 × 10^−^^8^
	GO:0006165	Nucleoside diphosphate phosphorylation	2.03 × 10^−^^9^
	GO:0046939	Nucleotide phosphorylation	2.03 × 10^−^^9^
	GO:0046434	Organophosphate catabolic process	3.67 × 10^−^^8^
	GO:0009135	Purine nucleoside diphosphate metabolic process	6.10 × 10^−^^9^
	GO:0009179	Purine ribonucleoside diphosphate metabolic process	6.10 × 10^−^^9^
	GO:0042866	Pyruvate biosynthetic process	3.02 × 10^−^^8^
	GO:0009185	Ribonucleoside diphosphate metabolic process	6.10 × 10^−^^9^
Cellular Component	GO:0005903	Brush border	0.043
	GO:1990584	Cardiac troponin complex	0.037
	GO:0043292	Contractile fiber	0.009
	GO:0044449	Contractile fiber part	0.008
	GO:0098850	Extrinsic component of synaptic vesicle membrane	0.020
	GO:0097452	GAIT complex	0.037
	GO:0031674	I band	0.037
	GO:0030016	Myofibril	0.009
	GO:0030017	Sarcomere	0.006
	GO:0005861	Troponin complex	0.015
Molecular Function	GO:0051373	FATZ binding	4.58 × 10^−^^5^
	GO:0016866	Intramolecular transferase activity	2.14 × 10^−^^4^
	GO:0016868	Intramolecular transferase activity, phosphotransferases	0.002
	GO:0000287	Magnesium ion binding	0.006
	GO:0004784	Superoxide dismutase activity	0.007
	GO:0016532	Superoxide dismutase copper chaperone activity	0.007
	GO:0031433	Telethonin binding	0.002
	GO:0016740	Transferase activity	9.75 × 10^−^^4^
	GO:0016769	Transferase activity, transferring nitrogenous groups	0.001
	GO:0009041	Uridylate kinase activity	0.007

**Table 4:** Gene ontology classification results of differentially expressed proteins of 24 h vs. 6 h PM.

	Term	Description	*p* Value
Biological Process	GO:0098656	Anion transmembrane transport	2.42 × 10^−5^
	GO:0006820	Anion transport	9.35 × 10^−5^
	GO:1905039	Carboxylic acid transmembrane transport	3.52 × 10^−4^
	GO:0015893	Drug transport	9.31 × 10^−6^
	GO:0051234	Establishment of localization	3.68 × 10^−4^
	GO:0034220	Ion transmembrane transport	6.68 × 10^−4^
	GO:0006811	Ion transport	2.37 × 10^−5^
	GO:0021675	Nerve development	3.52 × 10^−4^
	GO:1903825	Organic acid transmembrane transport	3.52 × 10^−4^
	GO:0006810	Transport	3.42 × 10^−4^
Cellular Component	GO:0031975	Envelope	4.82 × 10^−4^
	GO:0016021	Integral component of membrane	3.94 × 10^−10^
	GO:0031301	Integral component of organelle membrane	5.38 × 10^−4^
	GO:0031224	Intrinsic component of membrane	5.74 × 10^−10^
	GO:0098573	Intrinsic component of mitochondrial membrane	5.38 × 10^−4^
	GO:0044425	Membrane part	1.88 × 10^−4^
	GO:0005740	Mitochondrial envelope	3.47 × 10^−4^
	GO:0031966	Mitochondrial membrane	3.52 × 10^−4^
	GO:0031967	Organelle envelope	4.82 × 10^−4^
	GO:0031090	Organelle membrane	1.49 × 10^−4^
Molecular Function	GO:0022804	Active transmembrane transporter activity	6.62 × 10^−5^
	GO:0008509	Anion transmembrane transporter activity	7.26 × 10^−7^
	GO:0046943	Carboxylic acid transmembrane transporter activity	3.52 × 10^−4^
	GO:0015238	Drug transmembrane transporter activity	3.52 × 10^−4^
	GO:0015318	Inorganic molecular entity transmembrane transporter activity	8.75 × 10^−6^
	GO:0015075	Ion transmembrane transporter activity	8.75 × 10^−6^
	GO:0008514	Organic anion transmembrane transporter activity	2.42 × 10^−5^
	GO:0015291	Secondary active transmembrane transporter activity	2.42 × 10^−5^
	GO:0022857	Transmembrane transporter activity	3.15 × 10^−6^
	GO:0005215	Transporter activity	6.38 × 10^−6^

**Table 5:** KEGG pathway enrichment of differentially expressed proteins of 6 h vs. 0 h PM.

Pathway	Pathway Name	*p* Value
map00220	Arginine biosynthesis	0.037
map01230	Biosynthesis of amino acids	2.39 × 10^−5^
map01200	Carbon metabolism	7.47 × 10^−6^
map05204	Chemical carcinogenesis	0.005
map00270	Cysteine and methionine metabolism	0.006
map00982	Drug metabolism-cytochrome P450	0.009
map00051	Fructose and mannose metabolism	0.009
map00052	Galactose metabolism	0.037
map00480	Glutathione metabolism	0.006
map00010	Glycolysis/Gluconeogenesis	7.77 × 10^−9^
map04910	Insulin signaling pathway	0.029
map04213	Longevity regulating pathway-multiple species	0.009
map01100	Metabolic pathways	3.48 × 10^−7^
map00980	Metabolism of xenobiotics by cytochrome P450	0.015
map00030	Pentose phosphate pathway	0.004
map04146	Peroxisome	0.023
map00360	Phenylalanine metabolism	0.037
map00620	Pyruvate metabolism	0.001
map00500	Starch and sucrose metabolism	0.032
map00750	Vitamin B6 metabolism	0.007

**Table 6:** KEGG pathway enrichment of differentially expressed proteins of 24 h vs. 6 h PM.

Pathway	Pathway Name	*p* Value
map05010	Alzheimer’s disease	0.013
map04020	Calcium signaling pathway	8.00 × 10^−5^
map04022	cGMP-PKG signaling pathway	4.70 × 10^−4^
map05166	HTLV-I infection	0.002
map05016	Huntington’s disease	0.002
map04657	IL-17 signaling pathway	0.044
map04211	Longevity regulating pathway	0.044
map05144	Malaria	0.027
map04217	Necroptosis	0.039
map00190	Oxidative phosphorylation	0.006
map05012	Parkinson’s disease	6.80 × 10^−5^
map04742	Taste transduction	0.005
map00130	Ubiquinone and other terpenoid–quinone biosynthesis	0.014

**Table 7:** Validation of the differentially expressed proteins (PKM, TNNT1, COX5A, FIS1, COX4I1, and NDUFB11) by western blotting.

Accession	Gene Names	TMT (6/0 h)		WB (6/0 h)		TMT (24/6 h)		WB (24/6 h)	
		FC	*p* Value	FC	*p* Value	FC	*p* Value	FC	*p* Value
A5D984	PKM	0.64	0	0.64	0.0056				
Q8MKH6-2	TNNT1	1.47	2.15 × 10^−^^33^	1.57	0.0024				
P00426	COX5A	2.28	0.0001	1.72	0.0017				
Q3T0I5	FIS1					0.78	2.58 × 10^−^^6^	0.65	0.0031
P00423	COX4I1					0.49	3.34 × 10^−36^	0.61	0.0318
Q8HXG5	NDUFB11					0.87	0.0387	0.66	0.0216

FC indicates the fold change of each differentially expressed protein of 6/0 h PM or 24/6 h PM. TMT: tandem mass tags; WB: western blotting.

## Data Availability

The study did not report any data.

## Supplementary Materials

The following supporting information can be downloaded at: https://www.mdpi.com/article/10.3390/foods11020217/s1, Table S1: differentially expressed proteins were identified between 6 h and 0 h PM; Table S2: differentially expressed proteins were identified between 24 h and 6 h PM; Table S3: GO analyses of the differentially expressed proteins between 6 h and 0 h PM; Table S4: GO analyses of the differentially expressed proteins between 24 h and 6 h PM; Table S5: KEGG pathway analyses of the differentially expressed proteins between 6 h and 0 h PM; Table S6: KEGG pathway analyses of the differentially expressed proteins between 24 h and 6 h PM.
